# Statins and the risk of cirrhosis in hepatitis B or C patients: a systematic review and dose-response meta-analysis of observational studies

**DOI:** 10.18632/oncotarget.19611

**Published:** 2017-07-27

**Authors:** Yaqin Wang, Jianping Xiong, Meng Niu, Xiaowei Chen, Long Gao, Qirun Wu, Kechuang Zheng, Ke Xu

**Affiliations:** ^1^ Department of Interventional Radiology, The First Affiliated Hospital of China Medical University, Shenyang, China; ^2^ Department of Liver Surgery, Peking Union Medical College Hospital, Chinese Academy of Medical Sciences and Peking Union Medical College (CAMS & PUMC), Beijing, China

**Keywords:** statins, cirrhosis, fibrosis, meta-analysis

## Abstract

Hepatitis B and hepatitis C are leading causes of chronic liver disease, particularly cirrhosis. Recently, several studies have observed that statins have an inverse relationship with cirrhosis in hepatitis B or C patients. However, no published meta-analysis studied the protective effect of statins on cirrhosis. Thus, we conducted a systematic review and meta-analysis of published observational studies to better understand the relationship between statins and the risk of cirrhosis. Relevant studies were identified by searching PubMed, EMBASE, and ISI Web of Science for articles published before April 2017. The Newcastle-Ottawa Scale was used to evaluate the quality of the included studies. Six cohort studies, including 38951 cases of cirrhosis in 263573 patients with hepatitis B or C, were identified to investigate the relationship between statins and the risk of cirrhosis. The Newcastle-Ottawa Scale scores for the included studies ranged from 6 to 9, with four high-quality studies and only two of medium quality. The use of statins was associated with a significant 42% reduction in the risk of cirrhosis, without obvious heterogeneity. In addition, this protective effect was more obvious in Asian countries. Moreover, dose-response analysis suggested each additional 50 cumulative defined daily doses (cDDD) of statins decreases the risk of cirrhosis by 11% (odds ratio [OR] = 0.89, 95% confidence interval [CI] = 0.86–0.93, *p* = 0.001). In summary, statin use is associated with a decreased incidence rate of cirrhosis and is most pronounced in Eastern countries but also in Western countries.

## INTRODUCTION

Globally, approximately 130–170 million people have hepatitis C virus infection, which is equivalent to 2% of the world's population [1–3]. In addition, an estimated 350 million people – 5%–7% of the world's population – are chronic carriers of the hepatitis B virus [4, 5]; Moreover, hepatitis B and C are the leading causes of chronic liver disease, especially cirrhosis [6]. At least one-third of patients with cirrhosis have hepatitis B [7], and 10%-25% of patients with chronic hepatitis C will develop cirrhosis [8]. Cirrhosis is a primary cause of the global health burden. The number of cirrhosis deaths worldwide has increased from approximately 676000 in 1980 to over 1 million in 2010 [9].

Statins are a major cholesterol-lowering drug that has been used to prevent and treat various cardiovascular diseases. Recently, other potential benefits of statins have attracted increasing attention worldwide. For example, studies have indicated that statins can decrease the risk of some cancers, including prostate, colorectal, lung, breast and liver cancers [10–15]. In addition, other studies have reported that statins can significantly reduce portal pressure [16–18]. Additionally, researchers have observed an inverse relationship between statins and the risk of cirrhosis in hepatitis B or C patients. However, no published meta-analysis has investigated the effect of statins on the risk of cirrhosis. Thus, to better understand the relationship between statins and the risk of cirrhosis in hepatitis B or C patients, we conducted a systematic review and meta-analysis of the published observational studies.

## RESULTS

### Study selection and study characteristics

**Figure 1:**
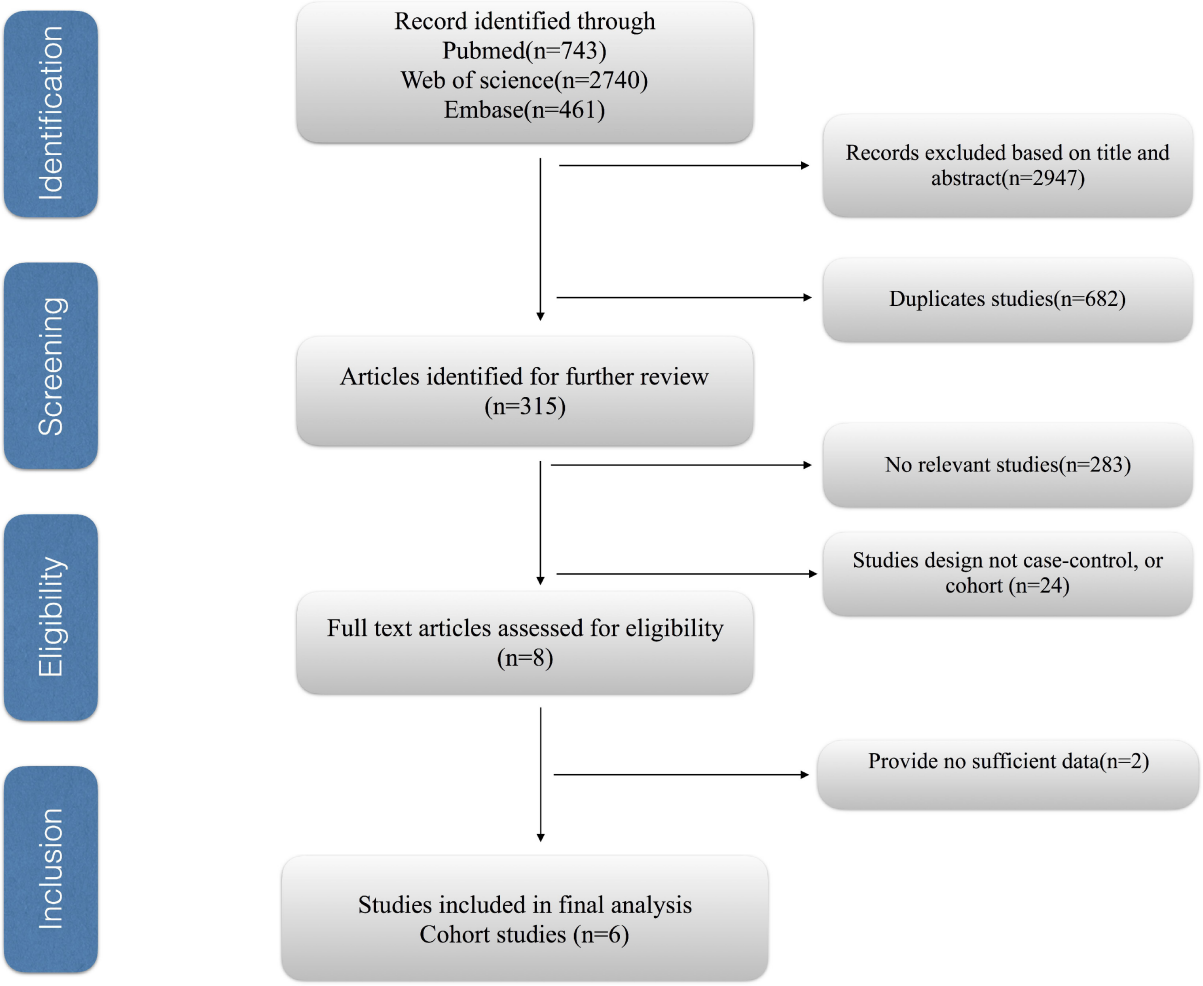
Process of selecting studies for the meta-analysis

Figure 1 shows the process of selecting studies for the meta-analysis. We obtained 3944 articles through the initial search, 682 of which were duplicated. We excluded a further 2947 studies based on a title and abstract review. Finally, two studies were further excluded due to providing insufficient information [19, 20], we identified six eligible observational articles for our meta-analysis [21–26].

The main characteristics of the included studies are listed in Table 1 [21–26]. Four of these were performed in the USA, and two were conducted in Taiwan. Overall, 38951 cases of cirrhosis in 263573 patients with hepatitis B or C included in these studies. The data were collected in the study from 1997 to 2014. The Newcastle-Ottawa Scale scores for included studies ranged from 6 to 9, with 4 high-quality studies and only two of medium quality (Table 3).

**Table 1:** Scores of the Newcastle-Ottawa scale for include studies. The asterisks represent a score (number of stars)

Study/Years of Publication	Country	No. Case/pesson-years	Follow	Sources of Controls	Subtype of study/ types of hepatitis virus	Exposure	Adjusted Factors	Comparison of Exposure Level (cDDD)	Adjusted OR/RR (95% CI)
Simon.2016 [23]	USA	1649/9135	2001–2014	population	cohort HCV	statin	age, sex, race, smoking history, alcohol abuse history, body mass index, diabetes, baseline FIB-4 score, metformin use, ACE inhibitor use, other lipid-lowering agent use, past completed anti-HCV treatment, attainment of SVR, and daily caffeine intake	28–89 VS never 89–180 VS never > 180 VS never	0.74 (0.59, 0.93) 0.71 (0.59, 0.88) 0.6 (0.53, 0.68)
Huang.2016 [23]	Taiwan	573/13086	1997–2009	population	cohort HBV	statin	age, gender, comorbidity index, hypertension, diabetes, hyperlipidemia, hepatocellular carcinoma, obesity, non-alcoholic fatty liver disease, aspirin use, diabetes medication, CHB treatment, non-statin lipid-lowering drugs, and triglyceride lipid-lowering drugs	28–90 VS never 91–365 VS never >365 VS never	0.85 (0.66,1.10) 0.47 (0.35,0.63) 0.20 (0.12,0.33)
Yang.2015 [21]	Taiwan	34273/226856	1997–2010	population	cohort HCV	statin	age, sex, urbanization, income, diabetes	28–83 VS never 84–365 VS never > 365 VS never	0.56 (0.35,0.89) 0.51 (0.34,0.77) 0.37 (0.20,0.71)
Simon.2015 [25]	USA	148/543	2010–2013	population	cohort	statin	established predictors of histological outcome, including body mass index, platelets and hepatic steatosis	statin use VS no statin use	0.31 (0.10,0.97)
					HCV				
Oliver.2016 [22]	USA	2265/5985	1999–2010	population	cohort HCV/HIV	statin	race, age, Deyo comorbidity score (without HIV), Era of HIV diagnosis, CD4+ cell count, BMI, diabetes, hypertension, HDL	statin use VS no statin use	0.68 (0.47 – 0.98)
Butt.2015 [26]	USA	43/7248	2002–2013	population	cohort HCV	statin	Age, sex, BMI, race, fibrosis, HCV-baseline level, diabetes- mellitus, behavioral factor	statin use VS no statin use	0.56 (0.50,0.63)

**Table 2:** Subgroup sensitive analyses for the effect of the use of statins on the risk of cirrhosis. cDDD, cumulative defined daily dose. RR, relative risk; CI, confidence interval

Subgroup	No. of studies	RR (95%CI)	*I^2^* value (%)	*P* value
All studies	6	0.58 (0.51, 0.64)	39.5	0.142
Geographic areas				
West	4	0.60 (0.53, 0.67)	42.7	0.155
East	2	0.48 (0.35, 0.61)	0	0.957
Study quality				
≥ 7	4	0.57 (0.50, 0.65)	51.6	0.102
< 7	2	0.54 (0.19, 0.89)	51.7	0.15
Patient with HBV or HCV				
HBV	1	0.49 (0.16, 0.83)	—	—
HCV	5	0.58 (0.50, 0.65)	49.5	0.095
Adjustment for confounders				
Alcohol intake				
Yes	2	0.64 (0.58, 0.69)	0	0.388
No	4	0.55 (0.48, 0.61)	6.7	0.359
Smoking				
Yes	1	0.64 (0.58, 0.70)	—	—
No	5	0.55 (0.49, 0.60)	0	0.504
Body Mass Index				
Yes	4	0.60 (0.53, 0.67)	42.7	0.155
No	2	0.48 (0.35, 0.61)	0	0.957
Diabetes				
Yes	5	0.58 (0.51, 0.65)	39.9	0.155
No	1	0.31 (0.10, 0.97)	—	—
Sensitive analyses				
Studies included in does-response analysis	3	0.57 (0.44, 0.69)	58	0.093
Fixed-effects vs random-effects model method				
Fixed-effects model	6	0.59 (0.55, 0.63)	39.5	0.142
Random-effects model	6	0.58 (0.51, 0.64)	39.5	0.142

**Table 3:** Main characteristics of the included studies

Study/Years of Publication	representativeness of exposed cohort	Selection of the non-exposed cohort	Determination of exposure	outcome not present at study start	Controlling the important factors or confounding factors	Assessment of outcome	Follow-up long enough for outcome to occur	Integrity of follow up	Total score
Simon.2016	*	*		*	**	*	*	*	8
Huang.2016	*	*	*	*	**	*	*	*	9
Yang.2015	*	*	*	*	*	*	*	*	8
Simon.2015		*	*	*	*	*	*		6
Oliver.2016	*		*	*	**			*	6
Butt.2015	*	*		*	**	*	*		7

RR, relative risk. OR, odds ratio. CI, confidence interval. SVR, sustained virological response. FIB-4, fibrosis 4 Score

ACE, angiotensin-converting-enzyme. HDL, high density lipoprotein. BMI, body mass index. HIV, human immunodeficiency virus. HBV, hepatitis B virus. HCV, hepatitis C virus.

### Overall results

**Figure 2:**
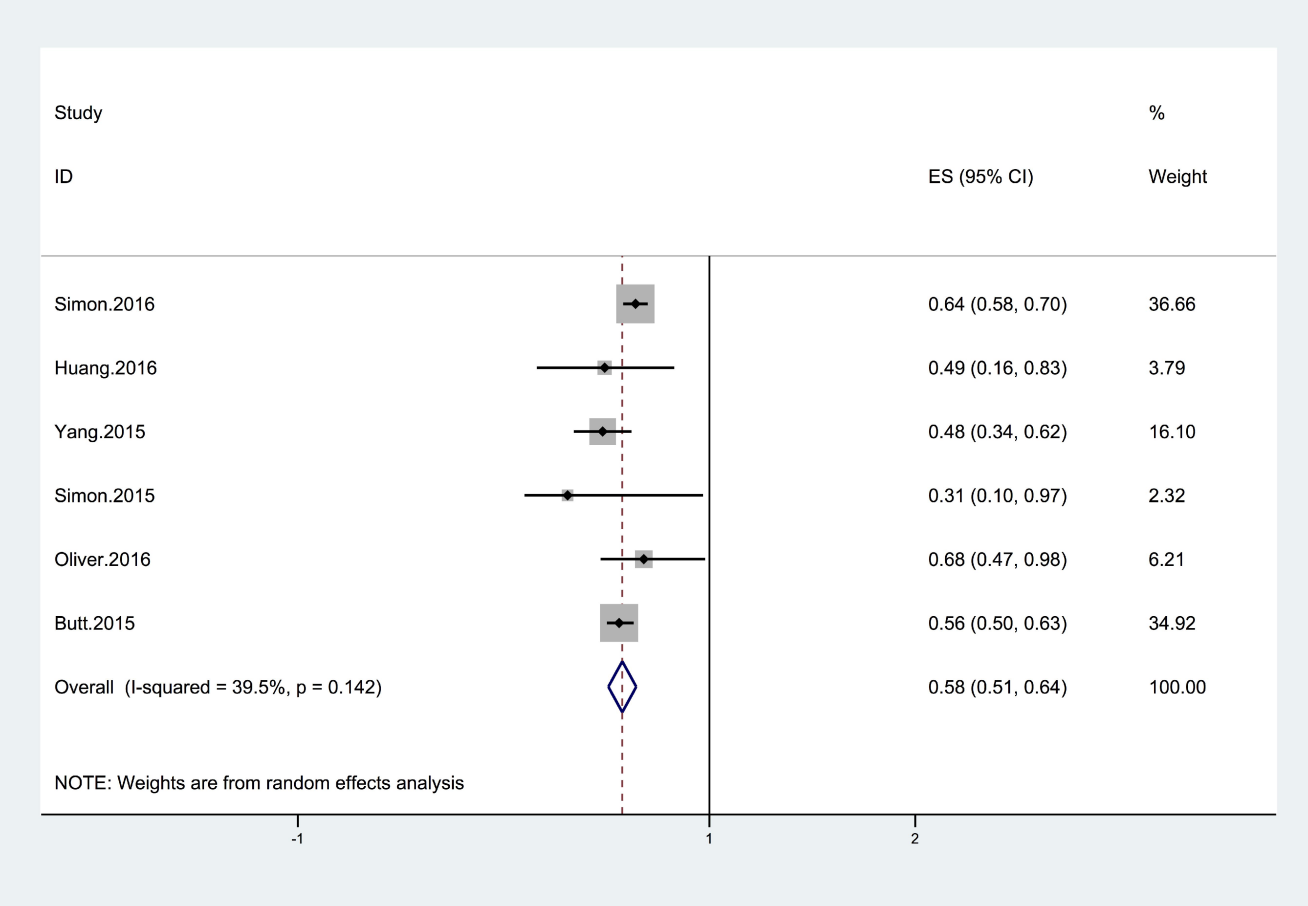
Forest plot showing the relationship between the use of statins and the risk of cirrhosis The points represent the risk estimate for each individual study. The horizontal lines represent the 95% confidence interval; the diamonds represent the summary risk estimate with 95% confidence interval. The area of square reflects the weight assigned to the study. CI, confidence interval. ES, effect size.

Six cohort studies [21–26] were identified to investigate the relationship between statins and the risk of cirrhosis in hepatitis B or C patients. We found that statin use was associated with a significantly lower risk of cirrhosis than never statin use (summary odds ratio [OR] = 0.58; 95% CI = 0.51–0.64). In addition, obvious heterogeneity was not detected in our study (I^2^ = 31.9%; *p* = 0.142). These results are shown in Figure 2.

### Subgroup and sensitivity analyses

**Figure 4:**
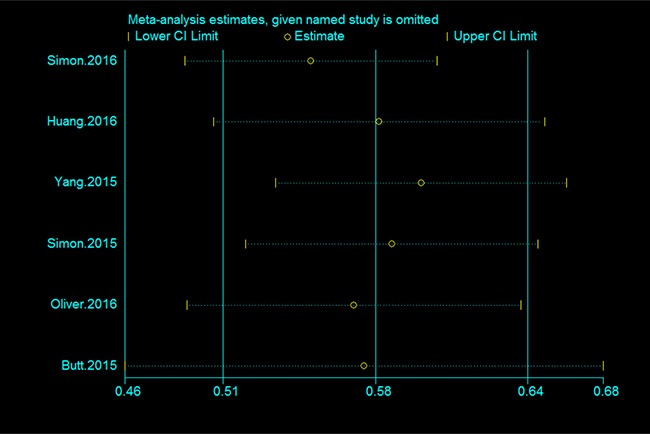
Sensitivity analysis of the association between statin use and the risk of cirrhosis

The results of subgroup analyses are shown in Table 2. When the analysis was stratified by geographic area, we found that use of statins was associated with a 40% decrease in the risk of cirrhosis in western countries, with a low heterogeneity. Moreover, the use of statins was associated with a significant 52% reduction in the risk of cirrhosis in the eastern countries; there was no heterogeneity observed within the group (Table 2). According to sensitivity analyses, despite excluding studies that were ineligible for dose-response analysis, the results for the relationship between statin use and cirrhosis remained stable (OR = 0.57; 95% CI = 0.44–0.69; I^2^ = 58.0%) (Table 2). In addition, the overall results were still steady when the pooling model was altered (fixed-effects model: OR = 0.59; 95% CI = 0.55–0.63 and random-effects model: OR = 0.58; 95% CI = 0.51–0.64) (Table 2). Sensitivity analysis was also performed to assess the effect of every study on the summarized estimate by sequentially excluding one study in one turn. In our meta-analysis, we detected no study could possibly affect the pooled risk estimate (Figure 4).

### Dose–response meta-analysis

**Figure 3:**
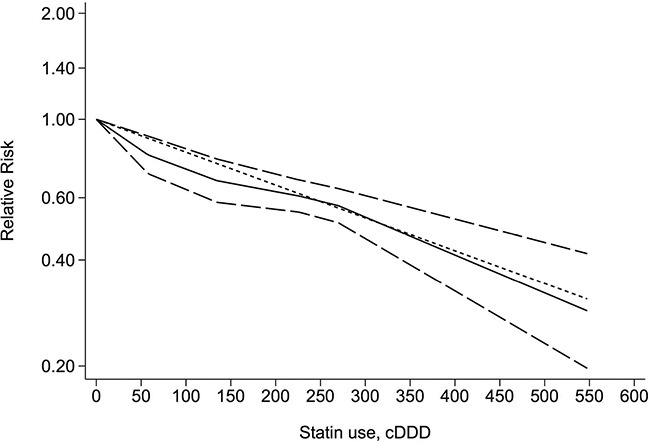
Dose-response relationship between statin use and the risk of cirrhosis The solid line and long dashed line represent the estimated relative risks and their 95% confidence intervals. The short dashed line represents the linear relationship. cDDD, cumulative defined daily dose.

Three studies [21, 23, 24] with a total of 36,495 patients with cirrhosis were eligible for the assessment of the dose–response relationship between statin use and the risk of cirrhosis in hepatitis B or C patients. When we used the restricted cubic splines model, we found that the concept of a nonlinear relationship between statin use and cirrhosis risk was rejected (p for nonlinearity = 0.2062) (Figure 3). However, we identified a linear relationship with a linear regression model (Figure 3). We found that each additional 50 cDDDs of statin decreased the risk of cirrhosis by 11% (RR = 0.89, 95% CI = 0.86–0.93, *p* = 0.001) (Figure 3).

### Publication bias

No testing for funnel plot asymmetry was performed because of the restricted number of included studies in the analysis (*n* < 10).

### Trial sequential analysis results

**Figure 5:**
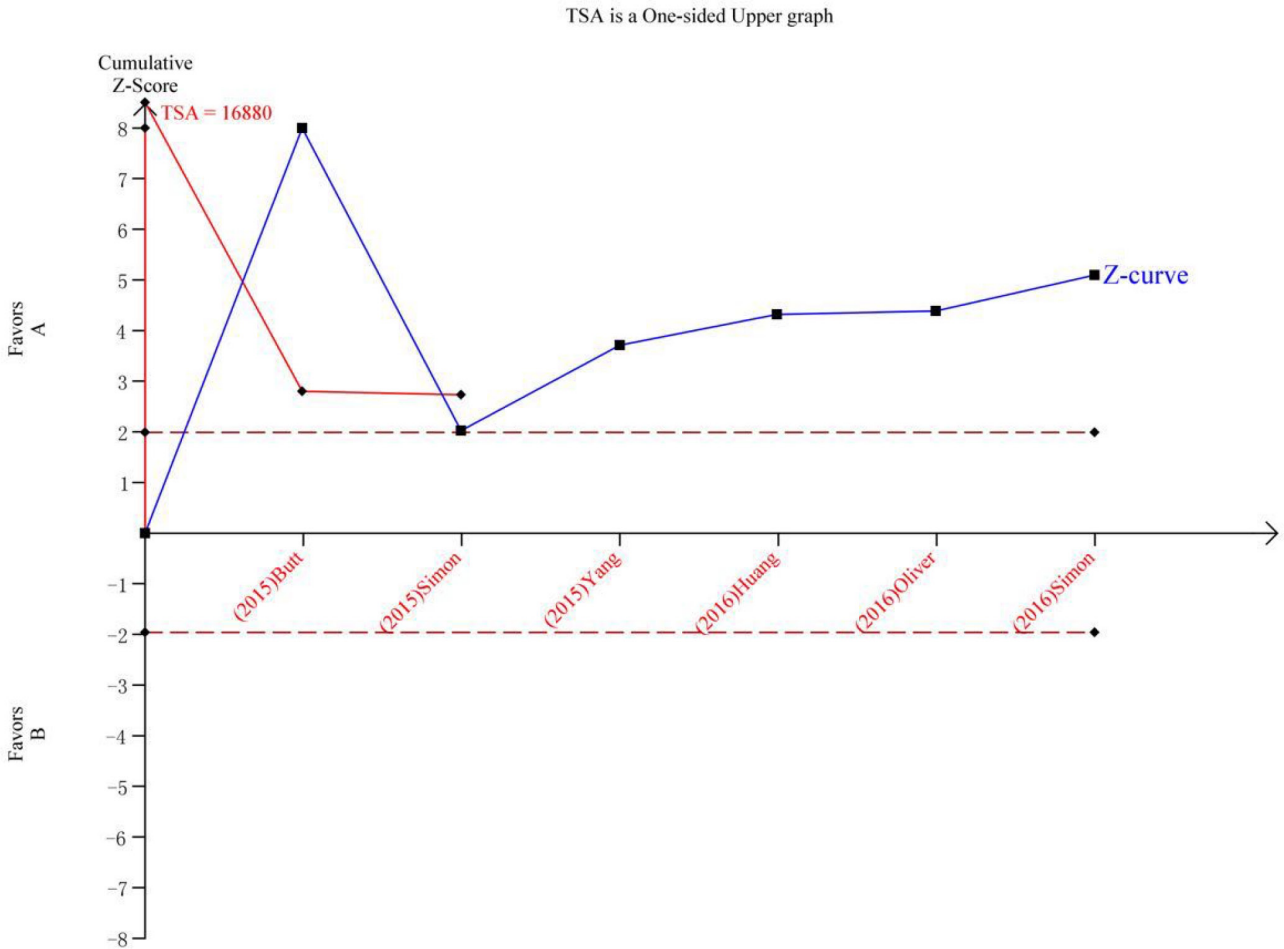
Trial sequential analysis of the association between between statin use and the risk of cirrhosis The Z-curve crosses the trial sequential monitoring boundary, and reach TSA information size. (**A**) Statin Use. (**B**) No Statin Use.

Trial sequential analysis (TSA) was performed for a more comprehensive assessment in our current meta-analysis. The cumulative Z-curve has crossed the monitoring boundaries already, demonstrating that our results were based on sufficient evidence. This finding revealed statin use were strongly associated with cirrhosis risk (Figure 5).

## DISCUSSION

Although published meta-analyses suggested a protective association between statin use and risk of hepatocellular carcinoma [27–29], no published meta-analysis studied the protective effect of statins on cirrhosis. To our knowledge, this is the first meta-analysis to investigate the relationship between statins and cirrhosis risk among patients with hepatitis B or C. We found that use of statins is associated with a 42% reduced risk of cirrhosis (OR = 0.58; 95% CI = 0.51–0.64). This protective effect is more obvious in Asian countries but is also found in Western countries. In addition, when we performed the dose-response analysis, a significant linear dose-response relationship was observed in our study rather than a nonlinear relationship. The study indicated that for every 50-cDDD increment in statin dose, the risk of cirrhosis was significantly decreased by 11%.

Our study only demonstrated an association between the use of statins and a reduced risk of cirrhosis; the data cannot establish a causative role for statins in this regard. However, if such a causative role is present, possible mechanisms could be the following. First, statins can inhibit the expression of fibrogenic cytokines such as connective tissue growth factor (CTGF), platelet-derived growth factor (PDGF) and transforming growth factor-b (TGFb), which play a critical role in the development of cirrhosis [30–32]. Second, statins can upregulate Kruppel-like factor 2 expression, which has beneficial effects on liver microcirculation and liver fibrosis in cirrhosis [33]. Third, statins may exert anti-HCV and anti-HBV activity by inhibiting cholesterol synthesis, HBV and HCV replication [14, 34, 35].

Our study has several strengths. First, it is the first to explore the dose–response relationship between statin use and the risk of cirrhosis in hepatitis B or C patients. Therefore, this may provide insight into the relationship between the use of statins and the risk of cirrhosis. Second, we performed subgroup and sensitivity analyses to determine which factors affect the risk. Third, most of the studies included in our meta-analysis were of high quality. Additionally, the studies included in the meta-analysis were cohort studies, which are not prone to generate recall and selection biases. All of these characteristics make the conclusions of our study more convincing.

There are several limitations that we must consider. First, there are many types of statins, including atorvastatin, cerivastatin, fluvastatin, lovastatin, pravastatin, rosuvastatin, simvastatin, and pitavastatin. However, we failed to obtain information about the use of other types of statin, which might have an influence on the final results. Second, the outcome that we observed was an association, which is subject to confounding bias. Although we considered several adjustment factors, there remain many potential adjustment factors that are unknown, such as the cholesterol level, the triglyceride level, or other over-the-counter drug use, which are closely related to the development of cirrhosis. In addition, we failed to obtain information about antiviral treatment in patients with HBV or HCV, which could have influenced the development of cirrhosis. Third, only 6 studies were included in our article, this puts the meta-analysis at high risk of publication bias. Additionally, Studies with different outcomes are combined which leads to the concern of heterogeneity in the meta-analysis. For example, in Simon 2015 [25], the outcome was “fibrosis progression”. However, in other studies, outcome was “development of cirrhosis”. Cirrhosis progression and development of cirrhosis are two different outcomes. Thus limiting us to generalize our findings to general populations. Finally, Simon 2016 [24] is a study of US veterans, a population that may or may not be generalizable to the normal population. Oliver 2016 [22] only included patients with HIV/HCV co-infection. It is known that progression to cirrhosis is threefold higher in co-infected than mono-infected patients. Thus, due to the different study designs and demographic characteristics inconsistency, the heterogeneity among studies acts as another potential limitation of this study.

In summary, our meta-analysis indicated that the use of statins is associated with a 42% lower risk of cirrhosis in hepatitis B or C patients. Moreover, this protective effect is more obvious in Asian countries. However, more prospective studies and basic research are still urgently needed to further validate the association between the use of statins and the risk of cirrhosis as well as the potential mechanisms.

## MATERIALS AND METHODS

### Data sources and search strategy

We searched published reports in the PubMed, EMBASE and Web of Science using the following keywords: (“statin* OR atorvastatin OR cerivastatin OR fluvastatin OR lovastatin OR pravastatin OR rosuvastatin OR simvastatin OR pitavastatin”) and (“cirrh* OR fibro*”). The initially relevant studies were identified up to April 2017, with no restrictions on the language of publication. We have extra data through contacting the original researchers.

### Eligibility criteria for study selection

The eligibility criteria were as follows: study design (case control or cohort); exposure factor statin and outcome cirrhosis or fibrosis; and odds ratio (OR)/risk ratio (RR) values and corresponding 95% confidence intervals (CIs) for different categories of statin use available or sufficient information provided to enable the calculation of these variables. If two studies reported the same data, we selected the study with the larger sample.

### Data abstraction and quality assessment

Two researchers (JPX and YQW) independently extracted the required information from the selected reports in a standardized manner. We collected the following information from each article: year of publication, first author's name and country of origin, study design (case control or cohort), number of participants (cases, controls, or cohort size), duration of follow-up, sources of controls, comparison of exposure levels, potential adjusted confounding variables, OR/RR values and 95% CIs for different categories of statin use. To assess the dose–response, we also collected the number of case and person-years for each category of statin use.

We used the Newcastle-Ottawa Scale [36] to evaluate the quality of included studies. We assigned quality categories based on the scores of each study. The categories were the following: high quality (score 7–9), medium quality (score 4–6) and low quality (score less than 4) [37]. We resolved discrepancies by consensus.

### Statistical analyses

We assessed the relationship between statins and cirrhosis using OR/RR values and the corresponding 95% CIs. When the results provided were for multiple groups with the use of statins with OR or RR values and corresponding 95% confidence intervals, we combined them to obtain a single OR/RR value and corresponding 95% CI [37]. We treated the hazard ratio as equivalent to the RR. We used the random effects model proposed by DerSimonian and Laird to quantify the relationship between the use of statins and risk of cirrhosis [38].

To enable the meta-analysis of the dose–response, we extracted the number of cases and person-years and RRs with variance estimates for at least three quantitative exposure categories from each study. If the studies did not provide these data, we required sufficient information to calculate them. For dose–response analysis, we used the midpoint of statin use in each category as the dose of statin use. If the highest category was open ended, we set the midpoint of the category at 1.5 times the lower boundary; if the lowest category was open ended, we set the lowest boundary at zero [39]. We obtained the dose-response results for a 50-cumulative-defined-daily-dose (cDDD) increment of statin use. The defined daily dose (DDD) is a dose unit for statins, and refers to “the assumed average maintenance dose per day for a drug used for its main indication in adults”; the cDDD refers to the sum of dispensed DDDs of any statins during exposure period. Additionally, we used restricted cubic splines with four knots at fixed percentiles (5%, 35%, 65%, and 95%) of the distribution to evaluate a potential nonlinear relationship between the use of statins and risk of cirrhosis [40]. A *p* value for the non-linear dose-response relationship was calculated by testing whether the coefficient of both the second and the third spline was zero [41]. Greenland and Orsini were the pioneers of this method [40, 42], and many subsequent studies have described it in detail [43, 44].

We used I^2^ to assess heterogeneity between studies and defined low, medium, and high heterogeneity as 25%, 50%, and 75%, respectively [45]. If p was less than 0.1, we assumed definite heterogeneity. Begg's test [46], Egger's test [47] and funnel plot have insufficient power when there are less 10 included studies [48].

We also performed subgroup analyses by geographic area, number of cases, study quality, and whether alcohol intake, smoking, body mass index or diabetes was adjusted for in the models. Sensitivity analyses were performed by changing the pooling model (random-effects model or fixed-effects model) and excluding studies that were not eligible for dose–response analysis. Sensitivity analysis was also performed to assess the effect of every study on the summarized estimate by sequentially excluding one study in one turn.

All statistical analyses were performed using STATA version 12.0 (Stata).

### Trial sequential analysis

Random error can mislead results in meta-analyses. The random error risk may increase remarkably because of multiple looks on accumulating evidence when new trials emerge [49]. To obtain a more comprehensive assessment of meta-analyses, TSA was conducted to control the risk of random error. In this meta-analysis, TSA was performed by maintaining a 95% CIs, a 20% relative risk reduction, an overall type-I error of 5%, and a statistical test power of 80%, which the required information size was calculated and the trial sequential monitoring boundaries was constructed [50]. If the cumulative Z-curve crossed the trial sequential monitoring boundary or exceeded the required information size, demonstrating that our results were based on sufficient evidence [51]. The trial sequential analysis software (TSA, version 0.9; Copenhagen Trial Unit, Copenhagen, Denmark, 2011) was performed in this study.

## Abbreviations

HBV: hepatitis B virus
HCV: hepatitis C virus
RR: relative risk
OR: odds ratio
CI: confidence interval
cDDD: cumulative defined daily dose
